# Detection of *DZIP1L* mutations by whole-exome sequencing in consanguineous families with polycystic kidney disease

**DOI:** 10.1007/s00467-022-05441-4

**Published:** 2022-02-24

**Authors:** Jens Michael Hertz, Per Svenningsen, Henrik Dimke, Morten Buch Engelund, Hanne Nørgaard, Anita Hansen, Niels Marcussen, Helle Charlotte Thiesson, Carsten Bergmann, Martin J. Larsen

**Affiliations:** 1 Department of Clinical Genetics, Odense University Hospital, J. B. Winsløws Vej 4, 5000 Odense C, Denmark; 2 Department of Clinical Research, University of Southern Denmark, Odense, Denmark; 3 Department of Cardiovascular and Renal Research, Institute of Molecular Medicine, University of Southern Denmark, Odense, Denmark; 4 Department of Nephrology, Odense University Hospital, Odense, Denmark; 5 Department of Pediatrics, Rigshospitalet, Copenhagen, Denmark; 6 Pediatric Clinic, Vestergade 21, Køge, Denmark; 7 Department of Clinical Pathology, Odense University Hospital, Odense, Denmark; 8 Department of Medicine, University Hospital Freiburg, Freiburg, Germany; 9 Limbach Genetics, Medizinische Genetik Mainz, Mainz, Germany

**Keywords:** ARPKD, Polycystic kidney disease, DZIP1L, Whole-exome sequencing, Primary cilia

## Abstract

**Background:**

Autosomal recessive polycystic kidney disease is a cystic kidney disease with early onset and clinically characterized by enlarged echogenic kidneys, hypertension, varying degrees of kidney dysfunction, and liver fibrosis. It is most frequently caused by sequence variants in the *PKHD1* gene, encoding fibrocystin. In more rare cases, sequence variants in *DZIP1L* are seen, encoding the basal body protein DAZ interacting protein 1-like protein (DZIP1L). So far, only four different *DZIP1L* variants have been reported.

**Methods:**

Four children from three consanguineous families presenting with polycystic kidney disease were selected for targeted or untargeted exome sequencing.

**Results:**

We identified two different, previously not reported homozygous *DZIP1L* sequence variants: c.193 T > C; p.(Cys65Arg), and c.216C > G; p.(Cys72Trp). Functional analyses of the c.216C > G; p.(Cys72Trp) variant indicated mislocalization of mutant DZIP1L.

**Conclusions:**

In line with published data, our results suggest a critical role of the N-terminal domain for proper protein function. Although patients with *PKHD1*-associated autosomal recessive polycystic kidney disease often have liver abnormalities, none of the present four patients showed any clinically relevant liver involvement. Our data demonstrate the power and efficiency of next-generation sequencing-based approaches. While *DZIP1L-*related polycystic kidney disease certainly represents a rare form of the disease, our results emphasize the importance of including *DZIP1L* in multigene panels and in the data analysis of whole-exome sequencing for cystic kidney diseases.

**Graphical abstract:**

A higher resolution version of the Graphical abstract is available as Supplementary information

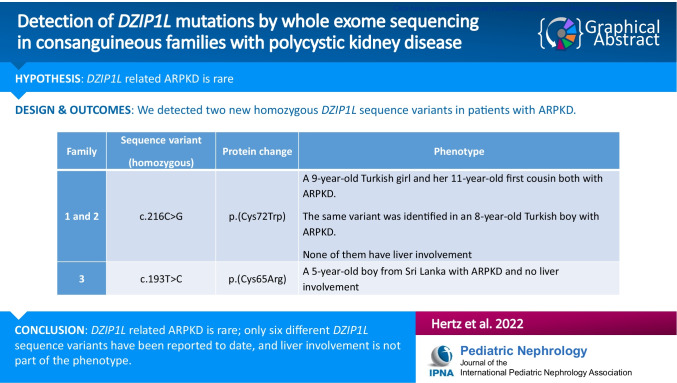

**Supplementary Information:**

The online version contains supplementary material available at 10.1007/s00467-022-05441-4.

## Introduction

Autosomal recessive polycystic kidney disease (ARPKD) is a cystic kidney disease with early onset, and an incidence of about 1 per 10–40,000 live births. It is characterized by cystic dilatation of the kidney collecting ducts and varying degrees of congenital hepatic fibrosis. Patients typically present with enlarged echogenic kidneys, hypertension, varying degrees of kidney dysfunction, and liver fibrosis (portal hypertension, splenomegaly, and esophageal varices) [1]. ARPKD is most frequently caused by sequence variants in *PKHD1*, encoding fibrocystin, but sequence variants in other genes related to primary cilia dysfunction may cause the same or clinically very similar phenotypes [2].

In 2017, Lu et al. [3] reported four different homozygous sequence variants in *DZIP1L*, located at 3q22.1, and encoding the basal body protein DAZ interacting protein 1-like protein (DZIP1L) in seven children with ARPKD from four consanguineous families. DZIP1L is located at the basal body of the primary cilium, and impaired function of DZIP1L is associated with a defect in the ciliary trafficking of the two major proteins for autosomal dominant polycystic kidney disease (ADPKD), polycystin-1 and polycystin-2. Since the first publication by Lu et al. [3], no other *DZIP1L* sequence variants have been reported.

We performed next-generation sequencing (NGS) in three consanguineous families comprising four children, all presenting with polycystic kidney disease, and thereby identified two different, not previously reported, homozygous *DZIP1L* sequence variants. Functional analyses of the p.Cys72Trp variant indicated disruption of a localization signaling or interaction domain of DZIP1L.

## Materials and methods

### Family history

#### Family 1

**Fig. 1:**
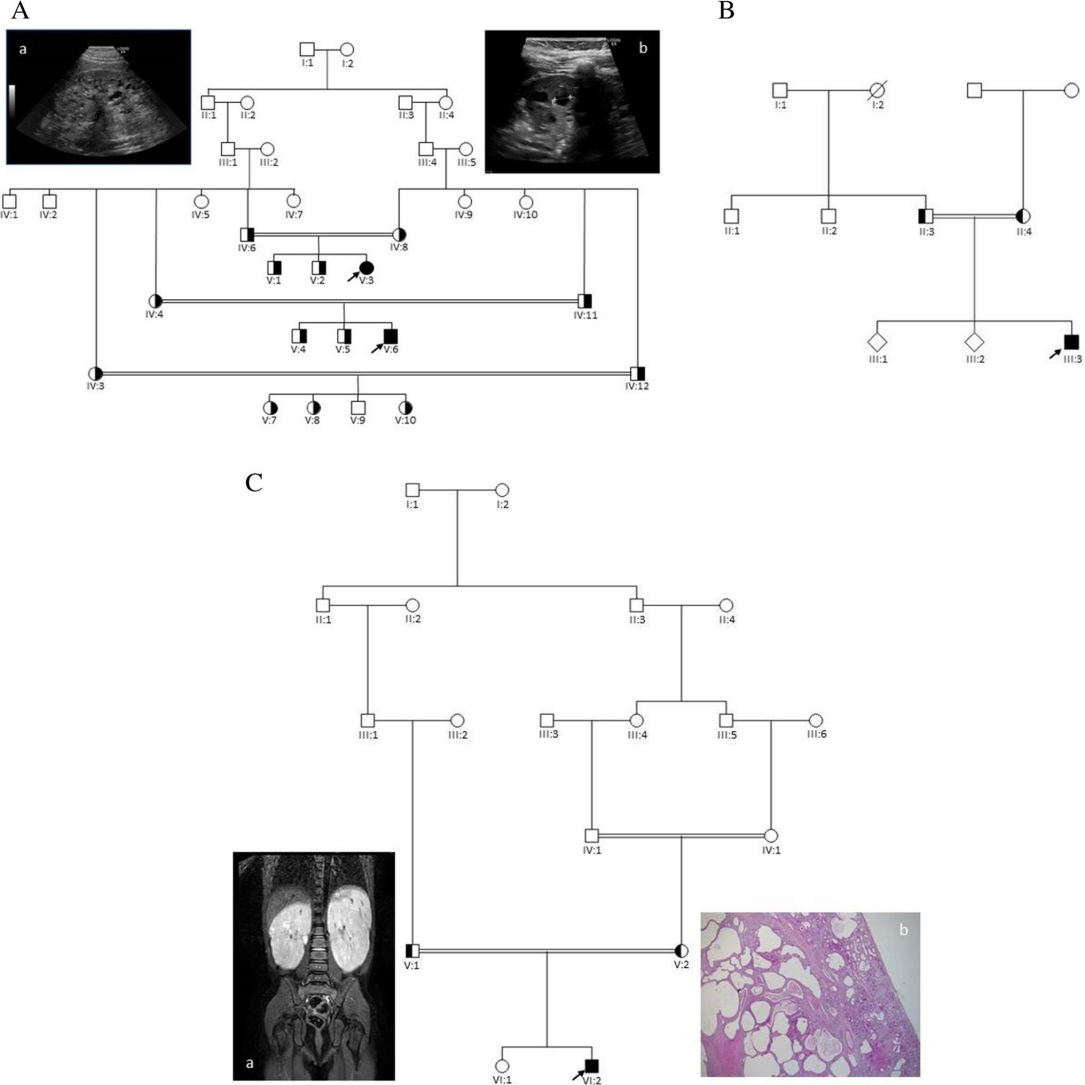
Pedigrees of the families. **A**: *Family 1*. The two affected children (V:3 and V:6) are both homozygous for the *DZIP1L* sequence variant: c.216C > G; p.(Cys72Trp). *a*: Ultrasound scan of the left kidney in V:3 and *b*: Ultrasound scan of the left kidney in V:6. **B**. *Family 2.* The proband (III:3) is homozygous for the same sequence variant as identified in *family 1*: c.216C > G; p.(Cys72Trp). **C.** *Family 3*. The proband (VI:2) is homozygous for the *DZIP1L* sequence variant: c.193T > C; p.(Cys65Arg). Filled symbols indicate homozygous individuals, and half-filled symbols indicate heterozygous relatives. *a*: MR-scan of the kidneys from VI:2 showing enlarged kidneys with multiple cysts. *b*: Histological sections from the right kidney in HE staining (VI:2): Cortical and medulla areas with cysts. Subcapsular, a small area shows a more normal structure. For details, see text. Arrows indicate the probands

The two affected children, V:3 and V:6 (Fig. 1A), are first cousins and the result of second cousin marriage (coefficient of inbreeding of 1/64) from a family of Turkish origin. The affected girl (V:3) was diagnosed with polycystic kidney disease at the age of 8 months after recurrent episodes of urinary tract infection. An ultrasound scan demonstrated bilaterally enlarged, echogenic kidneys with multiple small cysts and some larger cysts, and poor cortico-medullary discrimination. No cysts could be detected in the liver by ultrasound scan, and transient elastography using FibroScan® with the M probe (EchoSens, Paris) was normal. At the age of 9 years, she has increased blood pressure and reduced kidney function with an eGFR of 58 ml/min/1.73 m^2^. The affected boy (V:6) was diagnosed with polycystic kidney disease at the age of 6 years after a single episode of urinary tract infection. An ultrasound scan of the kidneys revealed comparable findings as seen in his affected female cousin. At his last visit to the Department of Pediatrics, at the age of 11 years, he was found to have bilaterally enlarged, echogenic kidneys with multiple small cysts and a few larger cysts up to 1.2 cm. The cortico-medullary discrimination was poor. No cysts could be detected in the liver by ultrasound scan, and transient elastography using FibroScan® with the M probe (EchoSens, Paris) was normal. His kidney function is reduced with an eGFR of 68 ml/min/1.73 m^2^. The parents are healthy with normal kidney function and no cysts in the kidneys could be detected by ultrasound scan.

#### Family 2

The male proband (III:3) is the third child of consanguineous parents (first cousins) originating from Turkey. He was diagnosed with polycystic kidney disease at the age of 8 years as an incidental finding. On ultrasound scan, kidneys were significantly enlarged (11 cm length, z score + 3.74) with multiple cortical and medullary cysts up to 8 mm in diameter. No cysts in other organs were detected, and no extrarenal symptoms or abnormalities were present. Psychomotor and intellectual development was unremarkable. By the age of 17 years, arterial hypertension developed and was treated with ACE inhibitors. Kidney function remained normal during adolescence until the last follow-up at the age of 18 years. No proteinuria or hematuria was present. Growth was normal (final height: 185 cm, 75th percentile). The proband’s father (II:3) had arterial hypertension and a history of urolithiasis, but no cysts in the kidneys could be detected by ultrasound scan. The paternal grandmother (I:2) had died of kidney failure of unknown cause, and two brothers (II:1 and II:2) with unremarkable kidney phenotype suffer from hearing loss of unknown origin. Both of them have healthy children.

#### Family 3

The male proband (VI:2) (Fig. 1C) is the result of a second cousin once removed marriage in a family from Sri Lanka, in which the parents of the proband’s mother (IV:1 and IV:2) are first cousins (coefficient of inbreeding of 1/64 for VI:2). He was diagnosed with polycystic kidney disease at the age of 5 years and underwent kidney transplantation aged 9 years. An enlarged right kidney of 15 × 6.5 × 6 cm was removed with several cysts < 1.5 cm in both cortex and medulla. By light microscopy, the cysts were of varying size and present in both cortex and medulla. Small subcortical areas showed a normal histological picture (Fig. 1C). The medulla was deformed mainly due to the various presences of cysts. All cysts were lined with a single layer of flattened cells. No cysts in the liver could be detected by ultrasound scan and transient elastography using FibroScan® with the M probe (EchoSens, Paris) was normal.

### Next-generation sequencing (NGS) approaches

In families 1 and 3, DNA samples from the affected children and both parents were subjected to whole-exome capture using SureSelect Human All Exon V5 (Agilent Technologies). Sequencing was performed on an Illumina HiSeq 2000 platform from Oxford Gene Technology (UK). A mean coverage of 120 × was obtained, and 95% of targeted bases was covered with a minimum of 20 × coverage. Raw reads were processed by using the Burrows–Wheeler Alignment tool version 0.7.15, and GATK Best Practice pipeline version 3.8–0 was used for variant calling. Annotation and filtering of variants were performed with VarSeq 1.4.6 (Golden Helix). Sequence variants were filtered according to autosomal recessive transmission pattern. Only splicing variants and rare coding variants were considered as potential pathogenic when the minor allele frequency was < 1%.

In family 2, we utilized our customized, targeted sequence capture approach previously described [4]. A customized NimbleGen (Madison, Wisconsin, USA) sequence capture library was designed that targeted all exons and an additional 35 bp of flanking intronic sequence for a very large number of genes associated with kidney diseases. DNA was enriched using the NimbleGen SeqCap EZ choice sequence capture approach and sequenced using an Illumina sequencing-by-synthesis technology. The average coverage was 500 × . Bioinformatic algorithms using a stepwise filtering process were developed for interpretation of generated NGS data.

### Sanger sequencing

All sequence variants were confirmed by Sanger sequencing using bidirectional sequencing with a BigDye® Terminator version 3.1 cycle sequencing kit and on a ABI3730XL capillary sequencer (Applied Biosystems).

### Plasmids

The human *DZIP1L* (NM_173543) cDNA clone was purchased from Origene. PCR amplified coding sequence of enhanced green fluorescent protein (EGFP) was inserted in the 5’ end of *DZIP1L* by Polymerase Incomplete Primer Extension (PIPE) [5], yielding N-terminal fusion of EGFP to human *DZIP1L*. The p.Cys72Trp mutation in *DZIP1L* was introduced by site-directed mutagenesis of c.216C > G. We did not succeed in creating the c.193 T > C. All constructs were verified by Sanger sequencing (Eurofins Genomics).

### Cells

Murine inner medullary collecting duct (IMCD3) cells (ATCC) and HEK293 cells (ATCC) were grown in DMEM:F12 cell culture medium supplemented with 10% fetal bovine serum and penicillin/streptomycin at 37 °C/5% CO_2_. Cells were seeded on coverslips and transfected with plasmids the following day using Metafectene as described by the manufacturer’s instructions (Biontex). Cells were grown in serum-free medium 24 h before imaging/antibody labeling.

### Immunofluorescence

Cells were fixed in formalin and permeabilized in 1% Triton X-100 (Sigma-Aldrich) in phosphate-buffered saline (PBS) for 10 min at room temperature. Cells were washed in PBS and incubated with mouse polyclonal anti-human DZIP1L protein antibody (1:500, H00199221-B01P, Abnova) overnight at 4 °C. Cells were washed in PBS and incubated with donkey anti-mouse AlexaFluor568 (1:500, Thermo Fisher Scientific) for 1 h at room temperature. Cells were washed and counterstained with DAPI (10 µg/ml, Sigma-Aldrich) for 5 min and mounted on glass slides.

### Fluorescence imaging

Confocal laser-scanning fluorescence microscopy (Olympus FV1000, Hamburg, Germany) was done using a × 20 (numerical aperture, 0.95) Olympus water immersion objective. Imaging of DAPI and EGFP was performed using sequential excitation from a laser at 405 and 488 nm with fluorescence emission monitored through appropriate acousto-optic tunable filter settings. Epifluorescence images were obtained using a × 40 objective on an Olympus BX51 mounted with a DP26 digital camera (Olympus) and were analyzed using Fiji (ver. 2.0.0-rc-43/1.51n) [6].

### Immunohistochemical staining of tissue for light microscopy and fluorescence microscopy

Kidney tissue obtained after nephrectomy due to kidney carcinoma was obtained from areas not affected by tumor growth. Specimens were immersion fixed in 10% formalin for 3 h, before dehydration and paraffin embedding. Paraffin-embedded tissue was stained as previously published [7]. Tissue was rehydrated and subjected to antigen retrieval in Tris-EGTA buffer (TEG, 10 mM Tris, 0.5 mM EGTA, pH = 9.0). 0.6% H_2_O_2_ and 50 mM NH_4_Cl in PBS were used to block endogenous peroxidase enzymes and free aldehyde groups, respectively. Sections were incubated with primary antibody overnight at 4 °C, washed, and incubated with secondary antibodies conjugated to horseradish peroxidase (HRP, DakoCytomation, Denmark). The DAB + Substrate Chromogen System was used to visualize HRP activity (K3467, DakoCytomation). Sections were counterstained with hematoxylin.

Double staining was done essentially as described above. After incubation with primary anti-DZIP1L mouse monoclonal antibody and secondary anti-mouse-HRP, a Cy3-coupled Tyramide Signal Amplification (TSA) substrate (TSA cyanine 3; Perkin Elmer, Waltham, MA) was added. Sections were then boiled in TEG and subjected to another round of immunolabeling using primary polyclonal goat anti-AQP2 (Santa Cruz Biotechnology, sc-9882) followed by Alexa Fluor-labeled 488 anti-goat secondary antibodies. An Olympus BX51 microscope was used for light and fluorescence microscopy.

## Results

### Family 1

Targeted NGS panel testing revealed no pathogenic sequence variants in either the autosomal dominant polycystic kidney disease (ADPKD) genes *PKD1* and *PKD2*, the ARPKD-related gene *PKHD1*, or in the nephronophthisis genes *NPHP11*, *NPHP13,* and *NPHPL1*, all found by SNP-array analysis to be positioned in regions of homozygosity (ROH) in the affected girl (V:3). Therefore, we searched for a causal mutation using a family-based whole-exome sequencing (WES) approach. Autosomal recessive inherited disease was suspected as the two affected first cousins were the only affected individuals in the family. Due to parental consanguinity, we searched for homozygous variants shared by the two affected first cousins. Only splicing variants and rare coding variants with a minor allele frequency of < 1% were considered. Only one homozygous missense variant in the *DZIP1L* gene fulfilled the filtering criteria (NM_173543.2:c.216C > G). This sequence variant is predicted to result in the replacement of cysteine with tryptophan at amino acid position 72 (p.Cys72Trp). Neither the specific variant nor any other missense change involving the cysteine residue at position 72 has been described in the literature or registered in public databases, including the Exome Variant Server, the Exome Aggregation Consortium (ExAC) database, and the gnomAD database. Evolutionary alignment for the *DZIP1L* variant was evaluated. Both the involved nucleotide and amino acid positions are highly evolutionarily conserved. Cysteine at amino acid position 72 was found to be highly conserved in selected vertebrae (chimp, macaque, rat, mouse, rabbit, dog, cat, cow, and frog). Functional prediction algorithms (MutationTaster, PolyPhen-2, and SIFT) were applied to evaluate the significance of the identified missense variant. All three prediction algorithms classified the *DZIP1L* missense variant as damaging (or functional, or disease-causing) with a probability of being damaging of 1 (MutationTaster and PolyPhen-2) and a SIFT score of 0.04. Substitutions with a SIFT score less than 0.05 are predicted to affect protein function. A CADD score of 22.3 was found. The *DZIP1L* variant was situated within a large region of homozygosity, where a region of approximately 10 Mb was shared by both affected cousins, indicating that the haplotype was inherited from a common ancestor.

The *DZIP1L* sequence variant was verified by Sanger sequencing in both affected children, and segregation analysis was carried out comprising the family's remaining members. Their parents and nine other healthy relatives were heterozygous carriers of the variant in line with autosomal recessive inheritance (Fig. 1A).

### Family 2

The same sequence variant identified in family 1 was also found in family 2. No other sequence variants were detected using the targeted NGS strategy as described.

Both families are of Turkish origin. While family 1 lives in Denmark, family 2 lives in Germany. By comparing various polymorphisms in *DZIP1L,* we found that all three affected family members harbor the same haplotype, indicating a common ancestor for the two families (data not shown).

### Family 3

Targeted NGS panel testing of the proband comprising *PKHD1*, *PKD1*, *PKD2*, and *HNF1B* was normal. Subsequent WES using a trio-based approach was therefore initiated. DNA from the proband was analyzed together with DNA from both parents. Due to parental consanguinity, we searched for homozygous variants in the proband and heterozygous in the parents. Rare coding variants and splicing variants were considered (minor allele frequency < 1%). Only one homozygous missense variant in the *DZIP1L* gene fulfilled the filtering criteria. A homozygous *DZIP1L* missense variant, c.193T > C; p.(Cys65Arg), was identified in the proband, VI:2 (Fig. 1C), for which both parents were found to be heterozygous. Prediction software indicates a functional effect of the variant with a CADD score of 25.7. Cysteine at amino acid position 65 is highly conserved in selected vertebrae (chimp, macaque, rat, mouse, rabbit, dog, cat, cow, and frog). No other disease-causing sequence variants in known polycystic kidney disease genes or others genes were detected.

The *DZIP1L* variants detected in the present study were subjected to American College of Medical Genetics pathogenicity evidence standards using the DECIPHER Consortium software [8]. Both homozygous variants reported were calculated to have a posterior probability of 0.975, thereby categorizing them as likely pathogenic.

### Expression and localization of wild-type DZIP1L and DZIP1L-Cys72Trp

We expressed fusion proteins of DZIP1L and DZIP1L-Cys72Trp and green fluorescent protein (GFP) in murine inner medullary collecting duct (IMCD3) cells by transient transfection. This approach created highly variable expression and localization in individual cells, and there were no obvious differences between the cellular localization of DZIP1L and DZIP1L-Cys72Trp (Suppl. Figure 1).

**Fig. 2:**
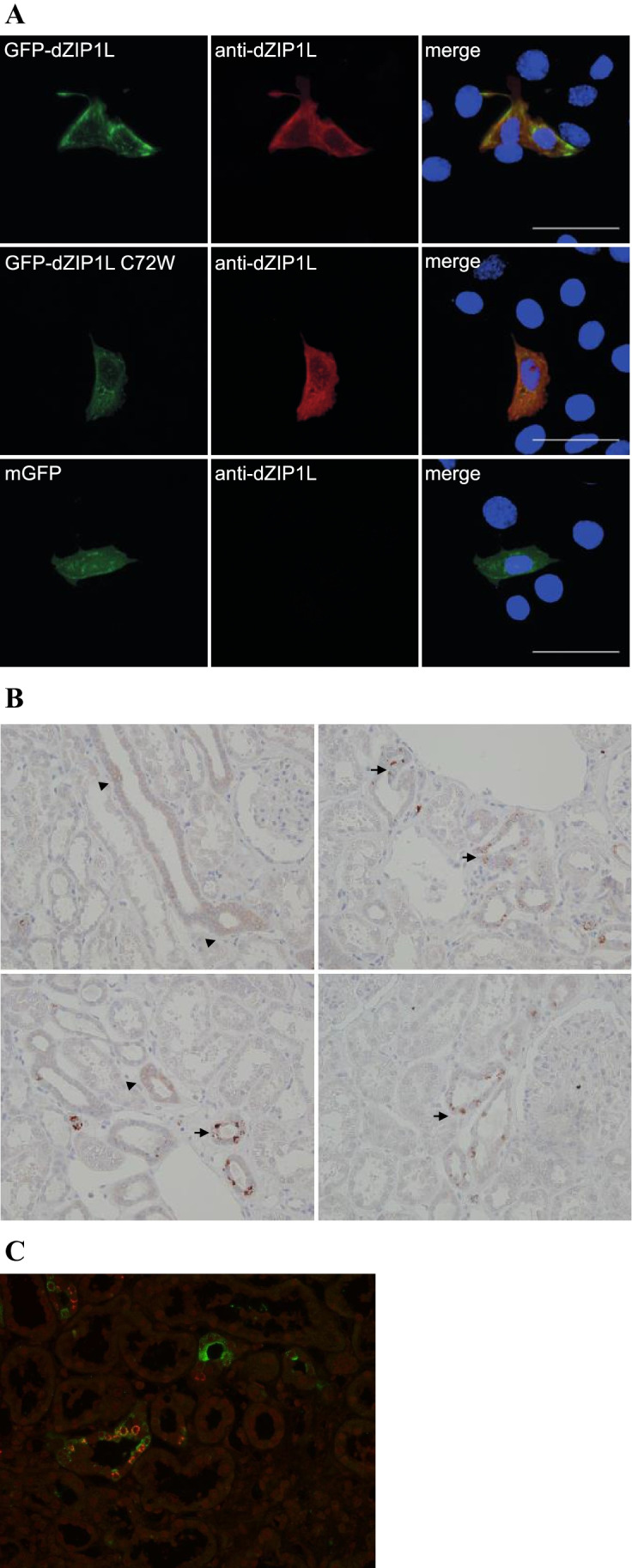
**A** HEK293 cells transiently transfected with GFP-DZIP1L WT, Cys72Trp and mGFP were labeled with an anti-DZIP1L antibody. The anti-DZIP1L antibody only labeled cells that were transfected with the DZIP1L constructs. Scale bar: 20 µm. **B** Normal adult human kidney samples were stained with the same anti-DZIP1L antibody. Arrows indicate strong punctuate staining observed in select cells. Arrowheads indicate weaker and more diffuse staining, mainly found in collecting duct cells. **C** Double staining with anti-DZIP1L (red) and AQP2 (green) shows that the strong punctuate staining is not restricted to AQP2 expressing cells in the collecting system

In an effort to analyze DZIP1L expression in human kidney samples, we first characterized the DZIP1L antibody (H00199221-B01P, Abnova) in HEK293 cells transiently transfected with GFP-DZIP1L and GFP-DZIP1L-Cys72Trp or membrane-tethered GFP. Using the antibody, staining was observed in cells transfected with GFP-DZIP1L and GFP-DZIP1L-Cys72Trp, but not membrane-tethered GFP (Fig. 2A). In the human kidney, staining predominated in the distal nephron (Fig. 2B). Two types of staining patterns could be distinguished. Intense staining could be found in punctuate form inside select cells along the distal nephron and collecting system (indicated by arrows), as well as more diffuse weaker staining in some collecting duct cells (Fig. 2B). Double staining with Aquaporin 2 (AQP2) expressed in principal and connecting tubule cells along with the collecting system showed that the intense DZIP1L staining was not restricted to AQP2 expressing cells in the collecting system alone, but was also localized to AQP2 negative cells, which likely represents intercalated cells (Fig. 2C).

## Discussion

In the present study, we detected two different, novel *DZIP1L* mutations in homozygous form in four patients with polycystic kidney disease from three consanguineous families (Table 1). This is only the second paper after the initial manuscript reporting *DZIP1L* mutations. So far, only six different *DZIP1L* variants, including the two present ones, have been reported (Table 2). Five of the reported sequence variants are located in exon 2, and four are missense mutations (**c.193T > C**; **p.(Cys65Arg)**, **c.216C > G**; **p.(Cys72Trp)**, c.269C > T; p.(Ala90Val), and c.273G > C; p.(Gln91His), of which the two present variants, shown in bold, are the most proximal ones, both affecting a cysteine residue. The remaining two variants described are a nonsense mutation (c.463C > T; p.Gln155*) located in exon 2 and a frameshift mutation in exon 7 (c.1061_1062del; p.Glu354Ala*39). We might therefore speculate that *DZIP1L* exon 2 is more sensitive to changes as a disease-associated region due to its protein function, given that the majority of variants are described there.

**Table 1:** Summary of the clinical and molecular genetic findings of the four affected individuals from the three families

Family	Individual (gender)	Parental consanguinity (coefficient of inbreeding)	Ethnicity	Sequence variant (all homozygous)	Protein change	CADD Phred score Phred score	Population allele frequency (gnomAD)	Phenotype
1	V:3 (female)	Yes (1/64)	Turkey	c.216C > G	p.(Cys72Trp)	22.322.3	Not detected	ARPKD. Diagnosed at the age of 8 months with bilaterally enlarged kidneys with increased echogenicity. Poor cortico-medullary discrimination. No liver cysts. Arterial hypertension and eGFR at 58 ml/min/1.73 m^2^ aged 9 years
1	V:6 (male)	Yes (1/64)	Turkey	c.216C > G	p.(Cys72Trp)	22.322.3	Not detected	ARPKD. Diagnosed at the age of 6 years with bilaterally enlarged kidneys with increased echogenicity and poor cortico-medullary discrimination. Multiple cysts up to 12 mm. No liver cysts. eGFR at 68 ml/min/1.73 m^2^ at the age of 11 years
2	III:3 (male)	Yes (1/8)	Turkey	c.216C > G	p.(Cys72Trp)	22.322.3	Not detected	ARPKD. Diagnosed at the age of 8 years with bilaterally enlarged kidneys with multiple cysts up to 8 mm. No liver cysts. Arterial hypertension from the age of 17 years
3	VI:2 (male)	Yes (1/64)	Sri Lanka	c.193 T > C	p.(Cys65Arg)	25.725.7	0.000003981(all) 0.00003266 (SA)	ARPKD. Diagnosed at the age of 5 years with multiple cysts up to 15 mm. No liver cysts. Right kidney removed (15 × 6.5 × 6 cm) and kidney transplantation at age 9 years

CADD = Combined Annotation Dependent Depletion; a tool for scoring the deleteriousness of single nucleotide variants. ARPKD = Autosomal Recessive Polycystic Kidney Disease, eGFR = estimated glomerular filtration rate. SA = South Asian

**Table 2:** The six DZIP1L sequence variants reported to date. All detected in homozygous form

Exon	Nucleotide change	Protein change	Domain affected	Reference
2	c.193T > C	p.(Cys65Arg)	-	Present family 3
2	c.216C > G	p.(Cys72Trp)	-	Present family 1 and 2
2	c.269C > T	p.(Ala90Val)	-	Lu et al. (2017) [3]
2	c.273G > C	p.(Gln91His)	-	Lu et al. (2017) [3]
2	c.463C > T	p.(Gln155*)	-	Lu et al. (2017) [3]
7	c.1061_1062del	p.(Glu354Ala*39)	Coiled-coil	Lu et al. (2017) [3]

Overall, *DZIP1L*-related ARPKD is a rare form of polycystic kidney disease. Lu et al. [3] sequenced 743 patients with a polycystic kidney disease phenotype and 805 with a nephronophthisis phenotype. They identified only the four described families with *DZIP1L* mutations among the 743 patients with suspected ARPKD or sporadic polycystic kidney disease (0.54%).

The kidney phenotype seen in ARPKD patients with *DZIP1L* mutations seems comparable to the phenotype seen in patients with mutations in *PKHD1* and characterized by enlarged echogenic kidneys with poor cortico-medullary differentiation and kidney impairment of varying degree. The clinical course is comparably mild in the three present families. The two affected first cousins in family 1, and the proband in family 2, all harboring the p.(Cys72Trp) substitution, have normal or only slightly reduced kidney function at the age of 9, 11, and 18 years. In contrast, the proband in family 3 with the p.(Cys65Arg) substitution had chronic kidney failure and underwent kidney transplantation aged 9 years. None of the present four patients show cysts in the liver, and transient elastography of the liver was performed in three of the four patients with a normal result. Among the seven cases with *DZIP1L* variants presented by Lu et al. [3], only one had mild signs of liver involvement in the form of hepatosplenomegaly. Therefore, congenital hepatic fibrosis might not be as frequent as in *PKHD1*-related disease. However, liver cysts and fibrosis may develop only later in life and may go clinically undetected for many years. The phenotypic variability may not solely be explained by the effect related to differences in the *DZIP1L* genotype, and other modifying genetic and non-genetic factors may have an impact on the phenotype as well.

Although the localization of GFP-DZIP1L and GFP-DZIP1L-Cys72Trp was variable in the IMCD3 cells, the wild-type form appeared to be more condensed into strongly fluorescent foci.

Our results demonstrate the power and efficiency of NGS-based approaches and emphasize the importance of including *DZIP1L* in multigene panels and in the data analysis of WES in patients with cystic kidney diseases.

## Supplementary Information

Below is the link to the electronic supplementary material.

Supplementary file1 (DOCX 898 KB)

Graphical Abstract 5441 (PPTX 44 KB)

## Data Availability

All data are available upon request by contact with the corresponding author.
