# Supplementary material for: Detection of DZIP1L mutations by whole-exome sequencing in consanguineous families with polycystic kidney disease
Source: Pediatr Nephrol. 2022 Feb 24;37(11):2657–65. doi: 10.1007/s00467-022-05441-4 (PMC9489574; doi:10.1007/s00467-022-05441-4)
Supplement: Supplementary file 1 — Supplementary file1 (DOCX 898 KB) [file 467_2022_5441_MOESM1_ESM.docx]

*Supplemental figure 1*:

IMCD3 cells transfected with GFP-DZIP1L and GFP-DZIP1L-Cys72Trp. Top panel: The wildtype DZIP1L showed different cellular localization even in cells exposed to the same transfection/plasmid reagents. Bottom panel: Examples of IMCD3 cells transfected with in GFP-DZIP1L (left) and GFP-DZIP1L-Cys72Trp (right).

*
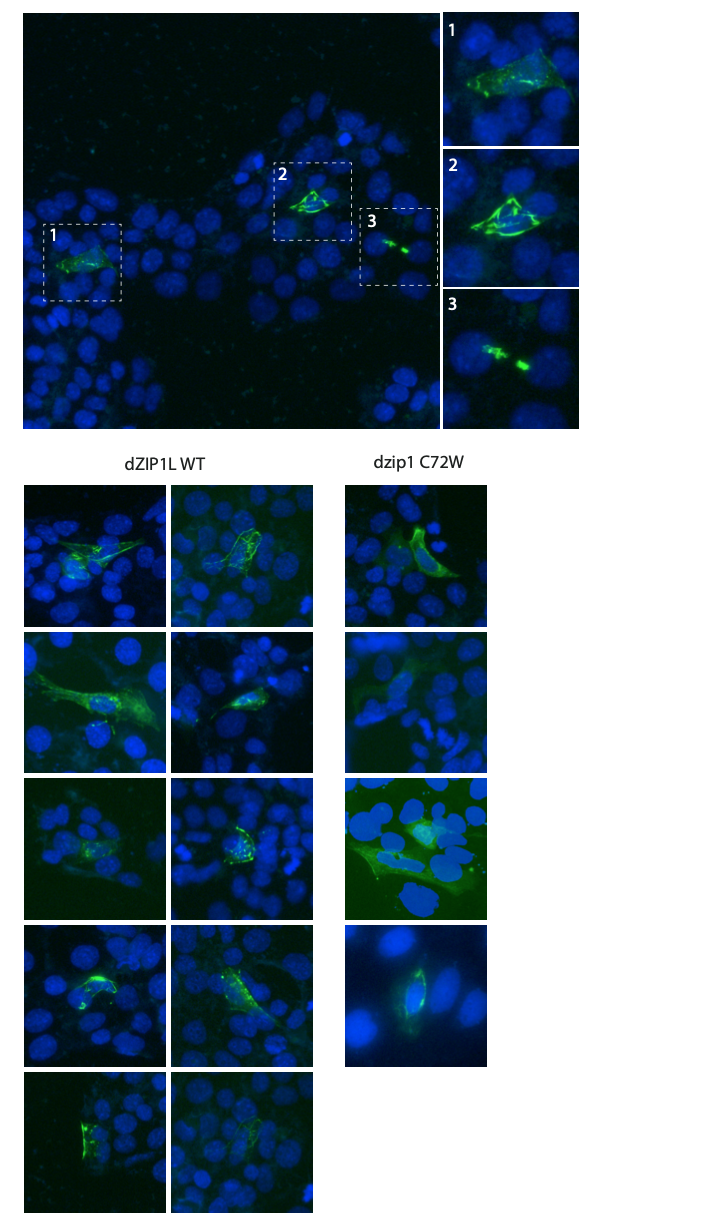
*
