# Supplementary material for: Detection of DZIP1L mutations by whole-exome sequencing in consanguineous families with polycystic kidney disease
Source: Pediatr Nephrol. 2022 Feb 24;37(11):2657–65. doi: 10.1007/s00467-022-05441-4 (PMC9489574; doi:10.1007/s00467-022-05441-4)
Supplement: Supplementary file 2 — Graphical Abstract 5441 (PPTX 44 KB) [file 467_2022_5441_MOESM2_ESM.pptx]

## Slide 1
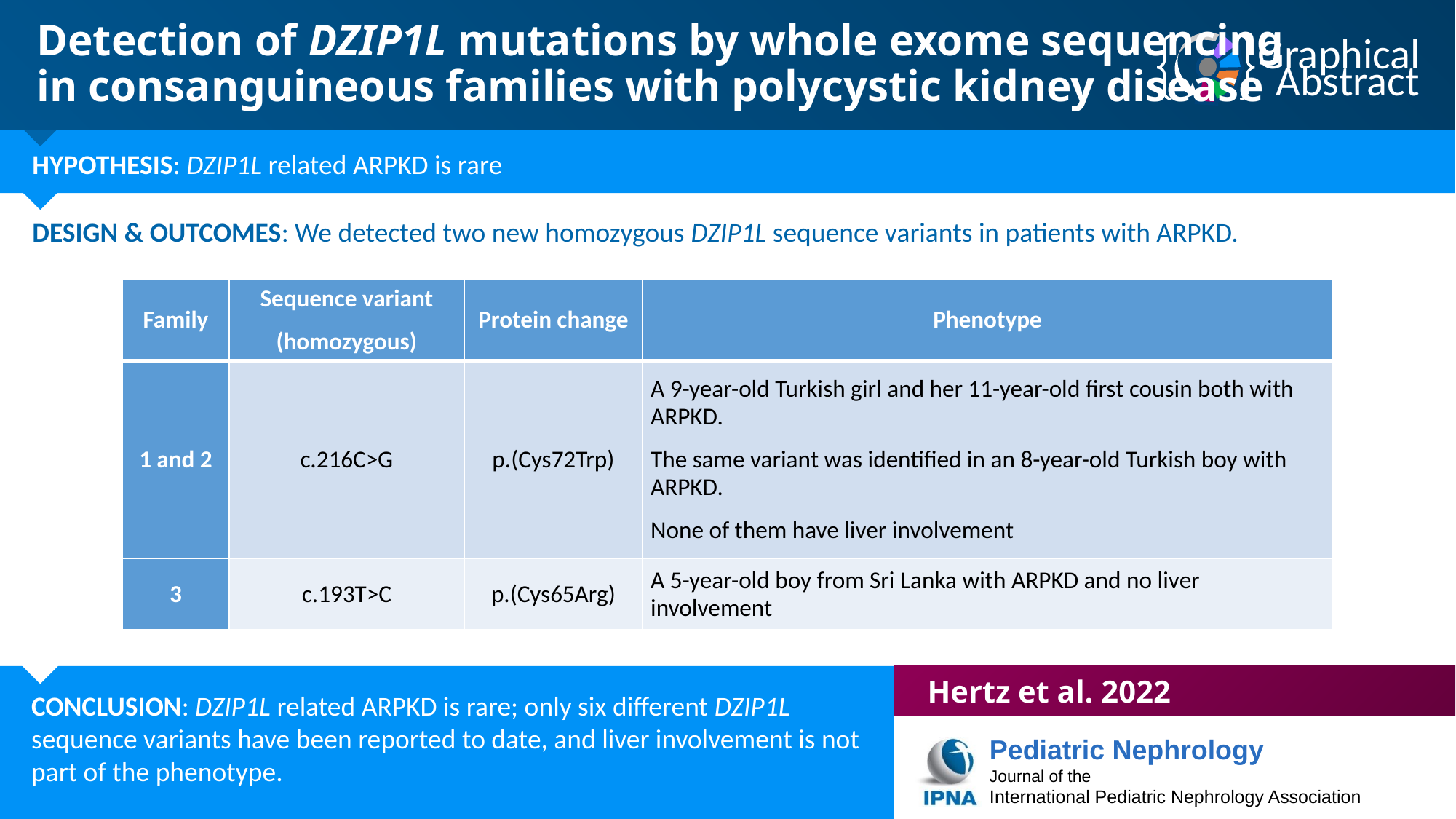

Detection of DZIP1L mutations by whole exome sequencing
in consanguineous families with polycystic kidney disease
HYPOTHESIS: DZIP1L related ARPKD is rare
DESIGN & OUTCOMES: We detected two new homozygous DZIP1L sequence variants in patients with ARPKD.
| Family | Sequence variant (homozygous) | Protein change | Phenotype |
| --- | --- | --- | --- |
| 1 and 2 | c.216C>G | p.(Cys72Trp) | A 9-year-old Turkish girl and her 11-year-old first cousin both with ARPKD. The same variant was identified in an 8-year-old Turkish boy with ARPKD. None of them have liver involvement |
| 3 | c.193T>C | p.(Cys65Arg) | A 5-year-old boy from Sri Lanka with ARPKD and no liver involvement |
Hertz et al. 2022
CONCLUSION: DZIP1L related ARPKD is rare; only six different DZIP1L sequence variants have been reported to date, and liver involvement is not part of the phenotype.
